# Nitrogen enrichment increases greenhouse gas emissions from emerged intertidal sandflats

**DOI:** 10.1038/s41598-020-62215-4

**Published:** 2020-04-21

**Authors:** Dallas J. Hamilton, Richard H. Bulmer, Luitgard Schwendenmann, Carolyn J. Lundquist

**Affiliations:** 1 Institute of Marine Science, University of Auckland, Auckland, New Zealand; 2 National Institute of Water and Atmospheric Research Ltd (NIWA), Hamilton, New Zealand; 3 School of Environment, University of Auckland, Auckland, New Zealand

**Keywords:** Biogeochemistry, Ecosystem services, Marine biology

## Abstract

Unvegetated, intertidal sandflats play a critical role in estuarine carbon and nutrient dynamics. However, these ecosystems are under increasing threat from anthropogenic stressors, especially nitrogen enrichment. While research in this area typically focuses on sediment-water exchanges of carbon and nutrients during tidal inundation, there remain significant gaps in our understanding of GHG (Greenhouse Gas) fluxes during tidal emergence. Here we use *in situ* benthic chambers to quantify GHG fluxes during tidal emergence and investigate the impact of nitrogen enrichment on these fluxes. Our results demonstrate significant differences in magnitude and direction of GHG fluxes between emerged and submerged flats, demonstrating the importance of considering tidal state when estimating GHG emissions from intertidal flats. These responses were related to differences in microphytobenthic and macrofaunal activity, illustrating the important role of ecology in mediating fluxes from intertidal flats. Our results further demonstrate that nitrogen enrichment of 600 gN m^−2^ was associated with, on average, a 1.65x increase in CO_2_ uptake under light (photosynthetically active) conditions and a 1.35x increase in CO_2_ emission under dark conditions, a 3.8x increase in CH_4_ emission and a 15x increase in N_2_O emission overall. This is particularly significant given the large area intertidal flats cover globally, and their increasing exposure to anthropogenic stressors.

## Introduction

Unvegetated soft sediment habitats have been shown to play a critical role in carbon and nutrient dynamics within estuaries and the coast^1–4^. These habitats are estimated to accumulate over 740,000 tonnes of carbon each year^5^, making significant contributions to reducing global carbon emissions. However, these ecosystems are under increasing threat due to stressors such as elevated nutrient loading sourced from anthropogenic activities such as agriculture and urbanization^6–8^.

Empirical measurements of fluxes on intertidal flats and the influence of nutrient enrichment on those fluxes have typically focused on the sediment-water column exchange of carbon and nutrients during tidal inundation (e.g.^2,3,9,10^). These submerged fluxes are subsequently used to infer fluxes during tidal emergence, either explicitly (e.g.^5^) or implicitly (e.g.^1^). However, key drivers that influence GHG (greenhouse gas) fluxes (specifically CO_2_, CH_4_, and N_2_O) on intertidal flats change significantly between tidal inundation and emergence, such as the benthic light climate^11^, temperature^12^, oxygen dynamics^12,13^, and macrofaunal activity^14,15^. If these drivers result in differences in GHG fluxes between submerged and emerged periods, upscaling from submerged fluxes to the full tidal cycle may result in inaccurate accounting of GHG budgets due to under- or over-estimates. Further uncertainties in GHG budgets for unvegetated intertidal flats occur due to the lack of understanding of the influence of nutrient enrichment on these habitats during tidal emergence, which could drive further differences in fluxes between submerged and emerged conditions.

The impact of nitrogen enrichment on estuaries is expected to worsen in the coming years (e.g.^7,16,17^). Consequently, reducing the uncertainty of estimates of the impact of nutrient enrichment on the flux of GHGs is an integral part of accurately quantifying GHG emissions from emerged intertidal flats. Here we investigate the impact of nutrient enrichment on GHG fluxes from emerged intertidal flats by manipulating sediment nutrient concentrations for several months at five sites on the North Island, New Zealand which vary in environmental characteristics. Three sites were selected in the Whangarei Harbour, with two sites in the Whangateau Harbour and the Raglan Harbour. Having three sites along an environmental gradient in the Whangarei Harbour allowed generalisations to be made for one estuary, while having a further two sites in different estuaries provided information on whether those generalisations could be applied to different estuaries. Further, we compare these values to data collected by other researchers during tidal submergence, to improve our understanding of how tidal state may impact GHG fluxes on intertidal flats.

We hypothesised nutrient enrichment will increase the emission of CO_2_, CH_4_ and N_2_O. We also hypothesised that that the flux of greenhouse gases from unvegetated intertidal flats will differ between periods of tidal emergence and periods of tidal submergence.

## Methods

### Study sites

**Figure 1:**
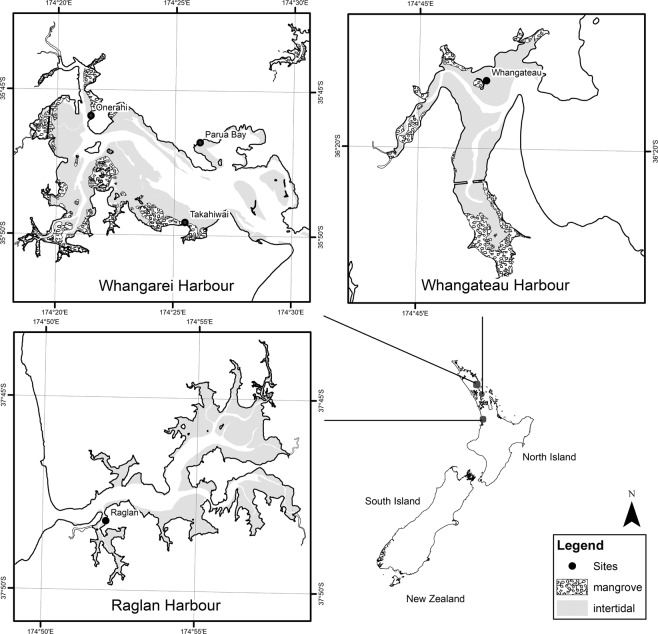
Study sites on the Whangarei, Whangateau, and Raglan Harbours in northern New Zealand.

Five sites across three estuaries were selected to span a range of environmental conditions (e.g. sediment mud content, organic matter content, chlorophyll concentrations, and macrofaunal community composition). Three of these sites were in the Whangarei Harbour, one was in the Raglan Harbour, and one was in the Whangateau Harbour (Fig. 1).

Whangarei Harbour is a large, unstratified estuary over 20 km long, covering an area of 104 km^2^ ^18,19^ and with a catchment area of 229 km^2^ ^20^. Mean depth in the harbour is 4.42 m^21^, with a mean tidal range of 1.73 m^18^. Approximately 28% of the harbour flushes each tide, and about half (54 km^2^) of the harbour area is intertidal^20,21^. Approximately two thirds of the intertidal area is composed of unvegetated, intertidal flats, with the remainder being mangrove forest and seagrass meadows, both of which have expanded in recent decades^22,23^. Land use in the catchment varies, with high proportions of pastoral agriculture and plantation forestry alongside urban areas and areas of native vegetation^20^.

Whangateau Harbour is a shallow estuary of approximately 9.2 km^2^, with little freshwater input^24^. The harbour has a high flushing rate, with up to 99% of the water being exchanged with each tide^24^, and 85% of the harbour is composed of intertidal flats^25^. The spring tidal range of the harbour is 2.2 m^26^. The harbour has a small catchment (~ 40 km^2^ in area), comprised primarily of native forest, but with areas of plantation forestry, livestock agriculture, horticulture, and urban use^26^.

Raglan Harbour is a drowned river valley of approximately 33 km^2^ area, of which ~70% is intertidal. The harbour has a maximum depth of 18 m, although channel depths closer to 5 m are more common. Raglan Harbour has a neap tidal range of 1.8 m and a spring tidal range of 2.8 m with 50–75% of the volume of the Harbour being exchanged each tidal cycle (at neap and spring tides respectively). The catchment is large (165 km^2^), and is dominated by agriculture and plantation forestry which have historically resulted in large inputs of sediment, with small areas of native forest and urban areas^27^.

### Nitrogen enrichment treatments

At each site, three nutrient enrichment treatments were performed: a high N treatment (600 g N m^−2^), a low N treatment (150 g N m^−2^), and a control with no enrichment. The application method and treatment doses were based on a previously tested method of nitrogen enrichment in New Zealand intertidal flats, which successfully resulted in long term nitrogen enrichment at treated sites for periods of >6 months^28,29^. Nitrogen fertiliser was placed in twenty 5 ×15 cm deep cores per m^2^, using a controlled-release, nitrogen-only, urea fertiliser (with no potassium, phosphorous, or trace elements). Fertiliser was inserted at a depth of 15 cm, with sediment cores removed, fertiliser added, and sediment returned to fill the remainder of the hole created by the core. This enrichment procedure was carried out 6 months prior to GHG flux measurements, in order to avoid impacts of the physical disturbance effects of enrichment and to measure the effect of chronic nutrient enrichment on those fluxes, which have been observed to occur at 3–6 months post nutrient enrichment using the same methodology^29^.

**Figure 2:**
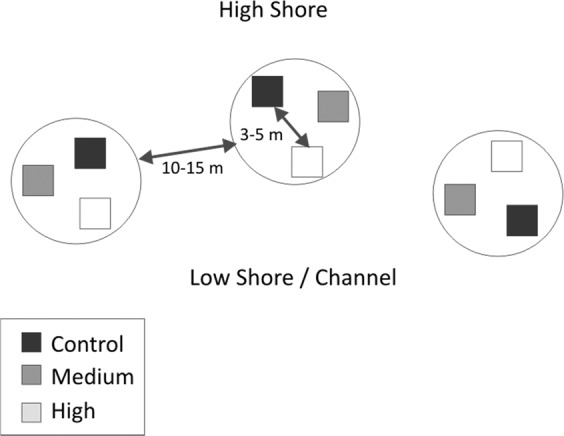
Example site layout of three replicate nutrient enrichment treatments. High Shore and Low Shore/Channel indicate proximity to terrestrial habitats, and toward open water, respectively.

Each treatment plot was 3 × 3 m in area, and plots were separated by at least 3 m from any other plot within each replicate block (Fig. 2). Treatments were replicated three times at each site in a randomized block design, for a total of nine plots per site. Replicate blocks were situated 10–15 m apart. All plots were located at similar tidal heights (approximately four hours of tidal emergence), with distances in tidal height between treatment plots negligible relative to the tidal range. Measurements carried out using static chambers in Light/Dark pairs separated by 0.2 m within each plot (for a total of 18 benthic chambers per site) and were at least 1 m from plot edges to minimise edge effects across the edge of treatment plots. Chambers were arranged in Light/Dark pairs to capture respiration rates (dark chambers) and the impact of photosynthesis/production (light chambers) on GHG emissions.

Nutrient enrichment at all sites was performed in April 2017, followed by sampling of GHG fluxes in November and early December 2017. Two of the five sites (Raglan and Whangateau) were re-treated with nitrogen fertiliser in December 2017, and a second round of sampling was carried out at these sites (and in the control treatments of the three sites in Whangarei) in June and July 2018 to investigate seasonal variation in GHG fluxes.

### Greenhouse gas flux measurement

GHG fluxes across the sediment-atmospheric interface were measured using 30 L, 0.25 m^2^ airtight benthic chambers. The measurement chambers were arranged in Light and Dark pairs, with one chamber having a clear lid and one an opaque lid. As soon as the receding tide exposed the study site, chambers were pushed 7 cm into the sediment. After approximately 10 minutes, the lids (and dark covers for dark chambers) were attached and sealed, and 10 minutes after that the first gas sample was taken. The 20 minute period between chamber placement and the start of flux measurements was chosen as a result of a preliminary experiment for this study^30^. As part of that experiment, the fluxes of GHGs were measured several times throughout the period of emergence at two sites with different environmental conditions, in order to determine the necessary sampling period for this study. The same light/dark paired *in situ* benthic chamber methods were used as those in this study, but with six gas samples collected from each chamber at approximately 25 minute intervals, instead of only collecting initial and final gas samples. This preliminary experiment showed no evidence of a large initial flux, with the flux of CO_2_ and CH_4_ over the first sampling period similar to that in the second and third period for each site, except for the CO2 flux in the light chambers at Onerahi. This provided strong evidence that there was not a high initial flux of GHGs in the chambers resulting from the physical disturbance of placing the chambers in the sediment, as any impact from this disturbance would have been expected across all treatments at all sites. Consequently, a 20-minute delay between the disturbance of placing the chamber and taking the initial measurement was used as a precaution but was considered adequate.

Initial 900 mL gas samples were collected from the chambers within ten minutes of sealing the chambers and stored in Tedlar bags. A final 900 mL gas sample was collected from each chamber immediately prior to tidal submergence at the site. The two samples in combination accounted for approximately 6% of the total 30 L volume of the measurement chamber. The period between the initial and final sampling of gases in the chambers ranged from 2–4 hours in length, depending on the magnitude of the tide, and the presence or absence of wind-driven waves that influenced the duration of tidal emergence/submergence.

Samples were kept in dark, insulated bins at ambient temperature and transported to the University of Auckland laboratory facilities for analysis. Gas samples were analysed within 24 hours of collection, using a Picarro G2508 Gas Analyser to determine the concentration of N_2_O, CO_2_, and CH_4_ gas in the samples. The detection and accuracy limits of the Picarro analyser are listed in the Supplementary Information.

The samples were collected twice for at each site, once in November/December 2017 for all nutrient enrichment treatments, and once in June/July 2018 for all enrichment treatments at the Whangateau and Raglan sites and for the Control treatment at the three sites in Whangarei. More regular sampling was not feasible, in part due to the unavoidable physical disturbance of sampling. It is likely that this disturbance, if it occurred more regularly, would influence the processes at those sites^31^, altering the fluxes and potentially obscuring the effect of prolonged nutrient enrichment on GHG fluxes.

### Flux calculations

Gas concentration measurements from the Picarro Gas Analyser were given in molar parts per million (ppm). To convert this to a standard gas flux (µmol m^−2^ hr^−1^), the Ideal Gas Law was used, calculating the molar concentration of a gas at the start and end of the gas sampling and the consequent change over time (Eq. 1).

1 $$flux=\frac{\left({G}_{F}\times \frac{{P}_{F}\times V}{R\times {T}_{F}}\right)-\left({G}_{I}\times \frac{{P}_{I}\times V}{R\times {T}_{I}}\right)}{A\times \Delta t}$$

where G = concentration of a given GHG in the chamber at the start (_I_) and end (_F_) of the measurement (ppm); P = atmospheric pressure at the start (_I_) and end (_F_) of the measurement (atmosphere); V = volume of gas in the chamber (L); R = Ideal Gas Constant of 0.0821 L atm mol^−1^ K^−1^; T = temperature in the chamber at the start (_I_) and end (_F_) of the measurement (K); A = area of sediment covered by chamber (m^2^); and Δt = time between the start and end of the measurement (hours).

### Site characteristics

While there are a multitude of environmental variables that may drive variation in the flux of GHGs, the following variables were selected with the aim of explaining the greatest possible proportion of variation in GHG fluxes with the limited resources available.

Temperature inside the chambers was recorded by 1-Wire i-button DS1921G-F5# automated loggers, located 10 cm from the edge of the chamber and 2 cm above the sediment surface. Photosynthetically Active Radiation (PAR) intensity was measured using two Odyssey Submersible PAR Loggers at each site.

Composite samples of five 2 cm deep, 2.5 cm diameter cores were collected from within 30 cm of the outer edge of each paired light and dark chambers to characterize sediment properties. To calculate pore water content, an approximately 15 g subsample was homogenised, weighed, freeze-dried for 8 days, and weighed again. The difference in weight between samples was calculated, and the proportion of the sample comprised from water expressed as a percentage. Chlorophyll a was analyzed by freeze drying an ~15 g subsample of homogenised sediment, producing approximately 5 g of dry sediment. Samples were boiled in 90% ethanol to extract chlorophyll a^32^. The extract was processed with a spectrophotometer (Shimadzu UV Spectrophotometer UV-1800). Samples were then acidified to separate phaeophytin from chlorophyll a^33^. The chlorophyll-a and phaeophytin content of the sediment is an indication of the microphytobenthic biomass that can occur at each site^34,35^. Due to resource constraints, more comprehensive measures of microbial activity could not be carried out. As such, the chlorophyll-a and phaeophytin content of the sediment are to be used implicitly as proxies for microbial activity.

Organic matter content was estimated using an ~5 g subsample of freeze-dried, homogenized sediment which was weighed, ashed at 450 °C for four hours^36^, and reweighed. Organic matter was calculated from the difference between dry and ashed weights. While attempts were made to measure the dissolved inorganic nitrogen concentration in the pore water at each site, due to analytical problems that data was unusable. As a result, the organic matter content of the sediment was used as a proxy.

Sediment grain size was measured using a Malvern Mastersizer 3000 (measuring particles 0.1–3500 µm in diameter). Samples were digested in 10% hydrogen peroxide until frothing ceased, rinsed with distilled water in a centrifuge, and run through the Malvern. The mud content was measured as the proportion of grains <63 µm in diameter.

Macrofaunal community characteristics have been found to be important factors influencing the flux of GHGs on temperate intertidal flats, with the abundance of large bioturbating bivalves being key drivers of this influence^15,37^. Consequently, the macrofaunal community characteristics were sampled once in summer and once in winter for each pair of Light/Dark chambers, using one 15 cm deep, 13 cm diameter sediment core immediately adjacent to the paired chambers (within the treatment plot). Cores were sieved on a 500 µm mesh. For the samples collected during summer, the material retained was preserved in 50% isopropyl alcohol and stained with Rose Bengal for subsequent analysis. All macrofaunal organisms observed were counted and identified to the lowest taxonomic level possible – in most cases species. Macrofaunal sampling in winter was limited to counts and measurements of bivalves >5 mm diameter (*Austrovenus stutchburyi*, *Macomona liliana*, and *Paphies australis*)*;* material was returned to the sampling site.

### Statistical analysis

The PERMANOVA + package for PRIMER v7 was used to quantify differences in the environmental variables measured at different sites and in different seasons, and to quantify differences in the flux of GHGs between and within sites, nutrient treatments, light exposure (i.e. Light or Dark chambers), and seasons.

Permanovas were carried out on Euclidian Distance Resemblance matrices, using the fixed factors of Site (Onerahi, Parua Bay, Takahiwai, Raglan, and Whangateau), Treatment (Control, Medium, or High nutrient enrichment), Light Exposure (Light or Dark), and Season (Summer or Winter) for the GHG fluxes.

For environmental variables, the factors of Site and Season were used. The highest order interaction that was significant at the p < 0.05 level was used to analyse differences in flux between groups of these factors. After an interaction was identified, post-hoc pairwise tests were carried out to identify which groups within those interactions were significantly different at the p < 0.05 level, and the direction of those differences.

## Results

### Site characteristics

The sites used in this study differed significantly in their environmental conditions (p < 0.05), exhibiting a gradient of environmental conditions (Table 1). Temperature, sediment mud content (<63 µm), sediment organic matter content, sediment porosity, and chlorophyll and phaeophytin concentration generally were lowest at Takahiwai, and highest at Raglan, with Onerahi, Parua Bay, and Whangateau exhibiting intermediate values (Table 1).

**Table 1:** Site characteristics (mean ± standard deviation) for each site and in each season where gas flux measurements were carried out.

	Onerahi				Parua Bay				Takahiwai				Whangateau				Raglan			
	Summer		Winter		Summer		Winter		Summer		Winter		Summer		Winter		Summer		Winter	
**Physical**																				
Temperature (°C)	22	(0.80)	20.8	(2.40)	21.1	(1.70)	19.6	(1.30)	17.5	(0.70)	20.7	(5.50)	23.2	(1.00)	20.8	(2.20)	22	(0.80)	13.2	(0.90)
Light intensity (umol photon m^-2^ s^-1^)	984		2323		1670		2604		426		3085		NA		1927		420		1192	
**Sediment**																				
Mud content (%)	5.9	(1.00)	9.1	(0.09)	7.8	(3.10)	12.3	(3.39)	1.5	(1.20)	1.7	(1.13)	3.5	(0.90)	9.7	(1.94)	17.5	(2.30)	20.4	(2.26)
Organic Matter (%)	1.8	(0.20)	1.2	(0.20)	2.2	(0.20)	1.7	(0.10)	1.4	(0.10)	1	(0.10)	1.4	(0.10)	1.1	(0.10)	3.9	(0.20)	2.7	(0.30)
Porosity (%)	0.49	(0.02)	0.49	(0.02)	0.45	(0.03)	0.45	(0.03)	0.47	(0.02)	0.47	(0.02)	0.47	(0.01)	0.47	(0.01)	0.53	(0.03)	0.53	(0.03)
**Microphytobenthos**																				
Chl a (dw mg g ^-1^ sediment)	8.8	(1.40)	5.8	(0.70)	6.1	(1.20)	7.8	(2.20)	8.4	(2.00)	8	(0.30)	10.7	(2.70)	12.9	(3.10)	17.2	(5.90)	15.4	(2.10)
Phaeophytin (dw mg g ^-1^ sediment)	3.2	(0.50)	3.1	(0.20)	2.6	(0.50)	3.5	(0.80)	3	(0.80)	3.6	(0.50)	3.2	(0.80)	5	(1.80)	9	(4.90)	7.1	(0.70)
**Macrofauna**																				
*Macomona liliana*	1.1	(1.00)	0.3	(0.50)	2.1	(2.10)	3	(0.00)	4.1	(2.90)	3	(0.90)	3.1	(2.60)	0.7	(0.70)	1.8	(1.80)	0.3	(0.50)
*Austrovenus stutchburyi*	3.9	(2.60)	0	(0.00)	14.8	(7.20)	5.3	(2.10)	12.2	(5.80)	7.3	(2.70)	37.4	(18.40)	10.7	(2.40)	55.6	(15.10)	18.6	(11.20)
Total bivalves	5	(2.80)	0.3	(0.50)	16.9	(7.90)	8.3	(2.10)	16.3	(7.80)	10.3	(3.60)	40.7	(20.50)	11.4	(2.70)	57.4	(15.60)	18.9	(11.30)

The sites differed in environmental conditions between summer and winter sampling, with sites generally having lower temperatures, organic matter content, and lower bivalve abundance in winter than in summer (Table 1), and higher mud content, chlorophyll, and phaeophytin concentration in winter (Table 1). However, the summer of 2016–2017 was an especially wet summer, with higher than average rainfall and lower than average daily temperatures. Also, the 2018 winter was an especially mild winter, with warm temperatures and clear skies for much of the season. This may have resulted in a smaller difference in environmental conditions between seasons than would usually be expected at these sites. Temperatures inside the chambers typically varied by 2–3 degrees in dark chambers and 3–4 degrees in light chambers over the duration of the chamber placement. The temperatures inside the chambers were on par with those outside the chambers, and did not reach temperatures higher than those that would be expected on the flats on a calm, fine day in the season the fluxes were measured.

### Greenhouse gas fluxes

At the non-enriched (control) sites, under Light conditions, 172 ± 76 µmol m^−2^ h^−1^ of CO_2_ was removed from the atmosphere, while under dark conditions 183 ± 56 µmol m^−2^ h^−1^ of CO_2_ was emitted. This gave an average net primary productivity (NPP) of 355 ± 132 µmol m^−2^ h^−1^. For this study, CO_2_ flux data is presented as separate light and dark fluxes instead of as NPP, as expressing the fluxes as NPP would not show the actual amount of CO_2_ being emitted (or taken up) by the intertidal flats, which is the key interest for this study. NPP can be inferred from the data already presented in this manuscript by simply subtracting the CO_2_ flux under dark conditions from the CO_2_ flux under light conditions.

No effect of Light or Dark treatment was observed for the CH_4_ or N_2_O fluxes, with emissions of 0.050 ± 0.14 µmol m^−2^ h^−1^ and 0.004 ± 0.0148 µmol m^−2^ h^−1^ respectively.

#### CO_2_

**Figure 3:**
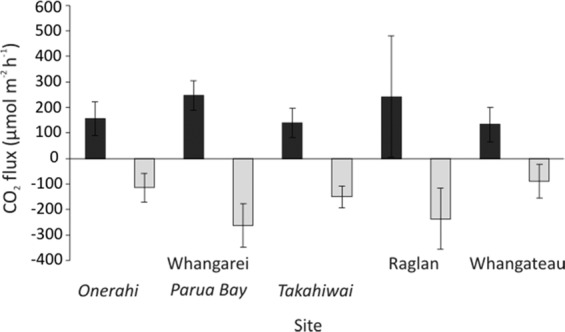
CO_2_ flux in the Control treatments for each site across both seasons sampled (mean ± standard deviation). Black bars represent Dark chambers, while light grey bars represent Light chambers.

For all nutrient treatments, at all sites, in all seasons, the chambers under Light conditions had significantly lower CO_2_ emissions (or higher uptake) than the chambers in Dark conditions (Fig. 3). The CO_2_ fluxes in Light conditions ranged from −0.1 to −670 µmol m^−2^ h^−1^ with a mean of −233 µmol m^−2^ h^−1^. In Dark conditions, the fluxes ranged from 2 to 890 µmol m^−2^ h^−1^ with a mean of 213 µmol m^−2^ h^−1^.

**Figure 4:**
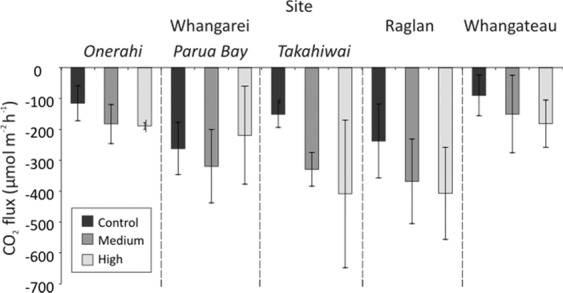
Mean CO_2_ flux averaged across seasons for each nutrient enrichment treatment (Control, Medium, High) at each site under Light conditions, mean ± standard deviation.

In Light chambers, the CO_2_ uptake in Control treatments was significantly lower (i.e. less CO_2_ emission) than in the Medium and High N enrichment treatments, which were not significantly different to each other (Table 2, Fig. 4). There were no significant differences due to nutrient enrichment on the flux of CO_2_ in Dark chambers (Table 2).

**Table 2:** PERMANOVA results comparing fluxes of CO_2_ as a function of different factors (site, nutrient enrichment treatment, light/dark conditions, and season).

Term	df	Pseudo-F	p(Perm)	Post hoc tests		
Site × nutrient	8	0.96203	0.4772			
Site × light	4	8.3302	0.0001			
Site × Season	4	1.0338	0.3937			
nutrient × light	2	6.6977	**0.0014**	**Light**	**Control**	
				C < M = H	L < D	
				**Dark**	**Medium**	
					L < D	
					**High**	
					L < D	
nutrient × Season	2	0.67088	0.5144			
light × Season	1	9.4367	0.002			
Site × nutrient × light	8	1.9591	0.0569			
Site × nutrient × Season	2	0.052131	0.9486			
nutrient × light × Season	2	1.2485	0.2904			
Site × light × Season	4	4.9939	**0.0015**	**Light, Summer**	**Onerahi, Summer**	**Raglan, Light**
				(RAG = TAK) < ONE < WHT,	L < D	S < W
				RAG < PB < WHT	**Parua Bay, Summer**	**Raglan, Dark**
				**Dark, summer**	L < D	W < S
				TAK < (RAG = WHT)	**Parua Bay, Winter**	**Whangateau, Light**
					D = L	W < S
					**Takahiwai, Summer**	**Whangateau, Dark**
					L < D	W < S
					**Raglan, Summer**	
					L < D	
					**Raglan, Winter**	
					L < D	
					**Whangateau, Summer**	
					L < D	
					**Whangateau, Winter**	
					L < D	

Significant effects (α = 0.05) are given in bold, and post-hoc pairwise tests are given for significant interactions. Significant interactions are prioritised over main effects, and three-way interactions over two-way interactions. C – Control nutrient treatment, M – Medium nutrient treatment, H – High nutrient treatment, RAG – Raglan site, TAK – Takahiwai site, ONE – Onerahi site, PB – Parua Bay site, WHT – Whangateau site, L – Light conditions, D – Dark conditions, S – Summer, W – Winter.

In Light chambers in summer, the CO_2_ flux at Raglan was significantly smaller (i.e. more negative) than any other site except Takahiwai, and the CO_2_ flux at Whangateau was significantly (p < 0.05) higher than any other sites (Table 2). In the Dark chambers during summer, Takahiwai had significantly smaller emissions of CO_2_ than any other sites, and there were no significant differences between any other sites. In winter, no significant differences were observed between sites (Table 2).

There was a seasonal effect on the flux of CO_2_ at Raglan and Whangateau, with the Dark chambers at Raglan and both the Light and Dark chambers in Whangateau having significantly smaller CO_2_ fluxes in winter than in summer (p < 0.05) (Table 2). In contrast, Light chambers in Raglan showed significantly smaller (i.e. larger negative) CO_2_ fluxes in summer than in winter (Table 2).

#### CH_4_

**Figure 5:**
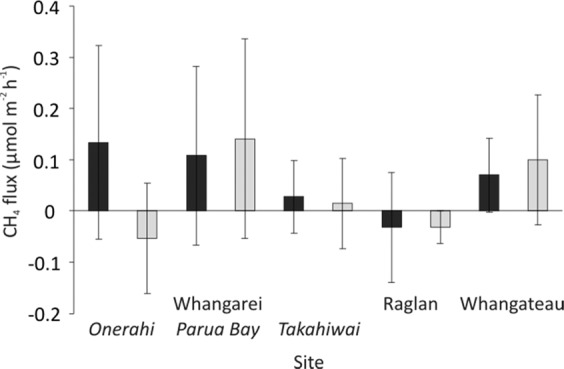
CH_4_ flux in the Control treatments for each site, averaged across seasons, mean ± standard deviation. Black bars represent Dark conditions, while light grey bars represent Light conditions.

The CH_4_ fluxes ranged from 0.82 to −0.65 µmol m^−2^ h^−1^, with a mean of 0.11 µmol m^−2^ h^−1^. There was a significant Site and Nutrient Treatment interaction, with significantly lower CH_4_ fluxes in the Control treatment than the High treatment at all sites except Parua Bay (p < 0.05). Overall, there were no significant differences in CH_4_ flux between Light and Dark conditions (Fig. 5, Table 3).

**Table 3:** PERMANOVA results comparing fluxes of CH_4_ as a function of different factors (site, nutrient enrichment treatment, light/dark conditions, and season).

Term	df	Pseudo-F	p(Perm)	Post hoc tests		
Site x nutrient	8	2.4779	**0.0173**	**Control**	**Onerahi**	
				RAG<WHT=PB,	C<H	
				TAK<WHT	**Takahiwai**	
				**High**	C<H	
				(ONE=PB)<RAG	**Raglan**	
					C<M<H	
					**Whangateau**	
					C<H	
Site x light	4	0.29948	0.8814			
Site x Season	4	1.0919	0.3636			
nutrient x light	2	1.8386	0.1717			
nutrient x Season	2	0.41859	0.6679			
light x Season	1	0.13413	0.7135			
Site x nutrient x light	8	1.5039	0.1709			
Site x nutrient x Season	2	1.4792	0.236			
nutrient x light x Season	2	1.1182	0.3264			
Site x light x Season	4	3.843	**0.0081**	**Light, Summer**	**Onerahi, Summer**	**Raglan, Light**
				ONE<TAK<RAG,	L<D	W<S
				ONE<PB=WHT	**Raglan, Summer**	**Raglan, Dark**
				**Dark, summer**	D<L	S<W
				RAG<(ONE=WHT)	**Raglan, Winter**	**Whangateau, Dark**
				**Dark, Winter**	L<D	W<S
				WHT<RAG		

Significant effects (α = 0.05) are given in bold, and post-hoc pairwise tests are given for significant interactions. Significant interactions are prioritised over main effects, and three-way interactions over two-way interactions. C – Control nutrient treatment, M – Medium nutrient treatment, H – High nutrient treatment, RAG – Raglan site, TAK – Takahiwai site, ONE – Onerahi site, PB – Parua Bay site, WHT – Whangateau site, L – Light conditions, D – Dark conditions, S – Summer, W – Winter.

**Figure 6:**
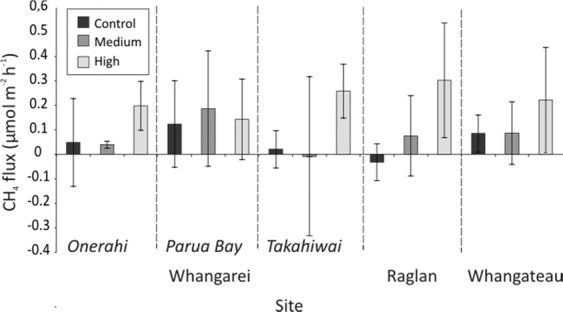
CH_4_ flux at each nutrient treatment at each site, mean ± standard deviation, with the measurements in Light and Dark conditions combined.

For the Control N enrichment treatment, the fluxes at Raglan were significantly smaller than the fluxes at either Parua Bay or Whangateau, and the fluxes at Takahiwai were also significantly smaller than the fluxes at Whangateau (Fig. 6, Table 3). There were no significant differences between sites for the Medium treatments, but for the High treatments Onerahi and Parua Bay both had significantly smaller CH_4_ fluxes than Raglan (Table 3). There were no significant differences between Control and Medium enrichment treatments at any sites except Raglan, and at Parua Bay there were no significant differences in CH_4_ flux between any enrichment treatments (Table 3).

#### N_2_O

**Figure 7:**
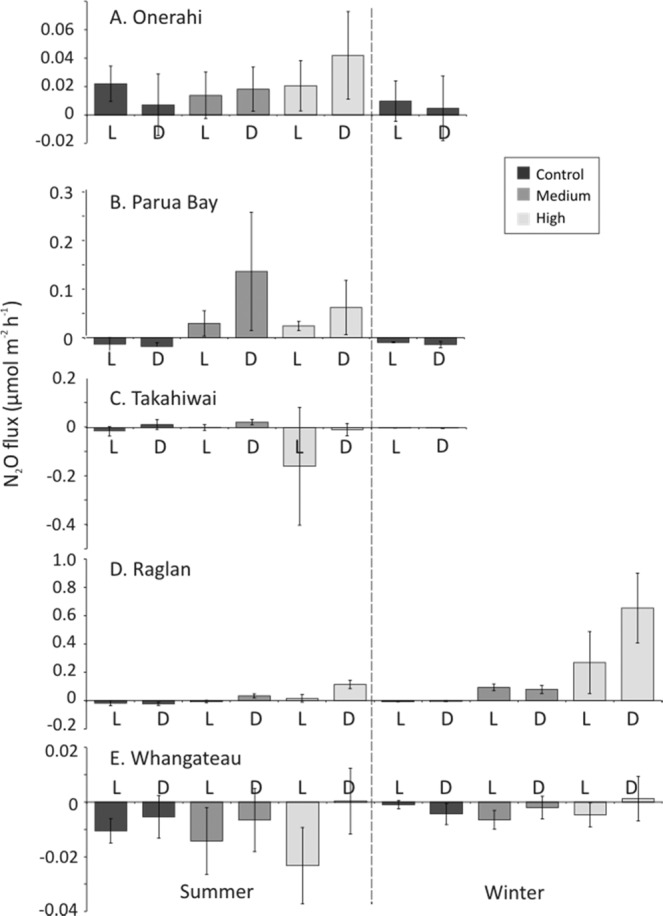
N_2_O flux (µmol m^−2^ h^−1^) within light (L) and dark (D) chambers for each of N enrichment treatments (Control, Medium, High), within each season (mean ± standard deviation). A. Whangarei Harbour, Onerahi; B. Whangarei Harbour, Parua Bay; C. Whangarei Harbour, Takahiwai; D. Raglan; E. Whangateau.

The N_2_O fluxes ranged from 0.84 to −0.44 µmol m^−2^ h^−1^, with a mean of 0.03 µmol m^−2^ h^−1^ (Fig. 7). There were two significant three-way interactions: a Site by Nutrient Treatment by Light Treatment interaction, and a Site by Nutrient Treatment by Season interaction (Table 4). There was a significant 4-way interaction between Site, Nutrient Treatment, Light Treatment, and Season (p < 0.05), though post-hoc pairwise tests showed no significant differences between any two groups in that interaction (Table 4).

**Table 4:** PERMANOVA results comparing fluxes of N_2_O as a function of different factors (site, nutrient enrichment treatment, light/dark conditions, and season).

Term	df	Pseudo-F	p(Perm)	Post hoc tests		
Site × nutrient	8	9.2892	0.0001			
Site × light	4	1.8507	0.1208			
Site × Season	4	6.5366	0.0003			
nutrient × light	2	4.0796	0.0183			
nutrient × Season	2	15.665	0.0001			
light × Season	1	0.67849	0.4221			
Site × nutrient × light	8	2.0812	**0.0462**	**Control, Dark**	**Parua Bay, Light**	**Raglan, High**
				(PB = TAK = RAG = WHT) < ONE	C < M < H	D < L
				**Control, Light**	**Parua Bay, Dark**	**Whangateau, High**
				PB < WHT < TAK, PB < ONE,	C < M < H	D < L
				(WHT = RAG) < TAK	**Raglan, Light**	
				**Medium, Dark**	C < M < H	
				WHT < (ONE = PB = RAG)	**Raglan, Dark**	
				**Medium, Light**	C < M < H	
				WHT < (ONE = PB = RAG)		
				**High, Dark**		
				WHT < (ONE = PB = TAK = RAG)		
				**High, Light**		
				WHT < (ONE = PB = TAK = RAG)		
Site × light × Season	4	0.58657	0.6637			
nutrient × light × Season	2	2.7951	0.0619			
Site × nutrient × Season	2	14.966	**0.0001**			
				**Control, Summer**	**Parua Bay, Summer**	**Raglan, Control**
				ONE < (OB = RAG = WHT)	C < (M = H)	S < W
				**Control, Winter**	**Raglan, Summer**	**Raglan, Medium**
				PB < RAG < TAK, PB < (RAG = ONE = WHT)	C < M < H	S < W
				**Medium, Summer**	**Raglan, Winter**	**Raglan, High**
				WHT < (ONE = PB = RAG), (WHT = TAK) < PB	C < M < H	S < W
				**Medium, Winter**		
				WHT < RAG		
				**High, Summer**		
				WHT < (TAK = ONE = PB), TAK < RAG		
				**High, Winter**		
				WHT < RAG		
Site × nutrient × light × Season	2	3.2761	**0.0455**	No significant pairwise differences at p < 0.05 level		

Significant effects (α = 0.05) are given in bold, and post-hoc pairwise tests are given for significant interactions. Significant interactions are prioritised over main effects, and three-way interactions over two-way interactions. C – Control nutrient treatment, M – Medium nutrient treatment, H – High nutrient treatment, RAG – Raglan site, TAK – Takahiwai site, ONE – Onerahi site, PB – Parua Bay site, WHT – Whangateau site, L – Light conditions, D – Dark conditions, S – Summer, W – Winter.

The N_2_O flux at both Parua Bay and Raglan increased with nutrient enrichment in both seasons, with the lowest fluxes in Control treatments, and the largest N_2_O fluxes in High nutrient treatments (Fig. 7b,d). Across all three nutrient treatments, and in both Light and Dark conditions, Whangateau had consistently smaller fluxes of N_2_O than other sites (Fig. 7e). Additionally, the chambers at both Raglan and Whangateau with High nutrient enrichment had significantly larger fluxes of N_2_O in the Light chambers than in the Dark (Table 4).

For the Site by Nutrient Treatment by Season interaction, nutrient enrichment was found to increase the flux of N_2_O in Parua Bay in summer (Fig. 7b), and in Raglan in both Summer and Winter (Fig. 7d). The only N_2_O fluxes that were significantly different between seasons were those in Raglan, with the N_2_O fluxes observed in Winter being significantly larger than those observed in Summer for all three nutrient treatments (Fig. 7d). In the Medium and High N enrichment treatments in both Summer and Winter, Whangateau consistently had significantly smaller fluxes of N_2_O (Fig. 7e), and Raglan had significantly larger fluxes of N_2_O (Fig. 7d, Table 4).

## Discussion

This study is the first to demonstrate that nitrogen enrichment is associated with an increase in CO_2_ uptake and CH_4_ and N_2_O emissions from emerged unvegetated flats, providing valuable new insights on the impact of nutrient enrichment on estuaries. This study also indicated that tidal emergence is associated with differences in the magnitude and direction of GHG fluxes when compared to fluxes measured during tidal submergence, improving our understanding of the role emerged intertidal flats play in estuarine GHG fluxes.

Nutrient enrichment of 600 gN m^−2^ was associated with an average of 1.65x increase in CO_2_ uptake under light conditions and 1.35x increase in the average CO_2_ emission in dark conditions (although the latter was not statistically significant). High N enrichment was also associated with a 3.8x increase in CH_4_ emission and a 15x increase in the emission of N_2_O under both light and dark conditions.

There have been several studies linking microphytobenthic activity to GHG emissions, with some estimates finding them responsible for as much as 50% of all estuarine primary production (i.e. CO_2_ flux) across all intertidal habitats^38^. These results suggest that nutrient enrichment enhanced microphytobenthic activity and resulted in a modest net reduction in CO_2_ emissions (driven by increased photosynthetic uptake) at our sites. However, this reduction in CO_2_ did not offset the significant increase in the emission of CH_4_ and N_2_O – both of which are likely also a result of increased microbial activity, as both methanogenesis and denitrification (the processes responsible for a majority of CH_4_ and N_2_O emissions respectively^1,4,39^) are controlled by microbial activity in the sediment^37^.

After accounting for the relative impact of each gas on the greenhouse effect by converting CH_4_ and N_2_O into CO_2_ equivalents (CH_4_ is 25x more potent than CO_2_ as a GHG; N_2_0 is 298x more potent than CO_2_ as a GHG^40^), an estimated average of the equivalent of 8.5 µmol m^−2^ h^−1^ of CO_2_ was emitted to the atmosphere from control sites under emerged conditions (allowing for both light and dark conditions). Nutrient enrichment resulted in an increase of 55% to 13.2 µmol m^−2^ h^−1^ of CO_2_ equivalents.

This study was carried out alongside another experiment measuring the flux of O_2_ (and by proxy CO_2_, as one flux can be converted to the other with an approximate 1: −1 ratio^41^) under submerged conditions at the same sites^42^. Under Light conditions, the submerged sand flats emitted an average of over 100 µmol CO_2_ m^−2^ h^−1^ ^42^. In contrast, emerged sediment from this study has taken up approximately 180 µmol CO_2_ m^−2^ h^−1^. These results suggest that, at least at these study sites, sediments may switch between being a source of CO_2_ during tidal submergence, to a sink of CO_2_ during tidal emergence. This may be due to the higher light intensity available at the surface of the sediment under emerged conditions^11,43^, allowing higher rates of photosynthesis by the microphytobenthos and lower rates of ventilation (meaning lower rates of CO_2_ release) by macrofauna such as bivalves under emerged conditions^44^.

While comparable submerged CH_4_ and N_2_O data was not collected at the same site, emerged fluxes in this study differ from submerged fluxes in other studies. Emerged CH_4_ fluxes in this study were on average 2.8x lower, and N_2_O fluxes on average 120x lower than submerged values reported elsewhere (Table 5). The differences in fluxes are consistent with the literature and likely driven by differences in environmental conditions. For example, in comparison to our study, CH_4_ and N_2_O emissions by Li *et al*.^2^ were approximately 3x and 5x higher, however sediment organic matter content was 8–8.5%, approximately 3–8x higher than our study. Sediment organic matter content is positively associated with rates of nutrient cycling, which drive increased N_2_O emissions^4,15,45^, and with microbial production of CO_2_ and CH_4_^46,47^. Macrofaunal community composition also varied significantly between sites in this study, and with other studies, such as Kang *et al*.^39^. Macrofauna influence the flux of CO_2_, CH_4_, and N_2_O through several processes, such as increasing CO_2_ emissions directly by increasing microbial respiration, and bioturbating the sediment^15,48,49^. Variability in the activity of macrofauna may also drive differences in GHG flux rates between tidal emergence and submergence. For example, both CH_4_ and N_2_O are produced within the sediment, CH_4_ via methanogenesis^1,50^ and N_2_O as part of the denitrification process^3,4,10^. The delivery of these gases to the sediment-atmosphere or sediment-water interface is accelerated by bioturbation (a process largely driven by bivalves on intertidal flats in NZ)^51^. Most bivalves, especially *A. stutchburyi* and *M. liliana* (the two most commonly occurring bivalves in this study), are known to reduce their activity during tidal emergence (e.g.^14,52^). This may decrease the delivery of GHGs from deeper in the sediment to the sediment surface. In addition, methodological differences may have contributed to differences between studies. For instance, Kang *et al*.^39^ have taken sediment in the field and incubated them in the laboratory for two hours, whereas our study used *in situ* chambers placed over the sediment surface for up to >3 hours, with minimal disturbance to the sediment column. Disturbance to the sediment column may impact O_2_ concentrations and expose anoxic sediment to oxygen^15,48^, enhancing GHG production^53–55^. The methods used in this study were established by a pilot study carried out in Hamilton^30^. Part of that preliminary experiment was to measure the flux of GHG several times throughout the period of emergence, in order to determine the ideal sampling period. The same *in situ* incubation methods were used, but with six gas samples collected at approximately 25-minute intervals, instead of only collecting initial and final gas samples from each chamber. There has been research identifying the potential influence of the ebb and flood tide on the emission of GHGs on intertidal flats through ‘tidal pumps’^39,56^. Based on this research, and on the results of the preliminary experiment described above^30^, GHG flux sampling for this experiment was conducted over both the incoming and outgoing tides to capture any ‘tidal pump’ impact on the emission of these gases. This provides a robust measurement of the net GHG flux on unvegetated intertidal flats over the whole emerged period, allowing the upscaling of these fluxes to larger time scales, such as days, weeks, or years.

**Table 5:** Mean GHG flux reported from different intertidal habitats including results from this study and the corresponding experiment during tidal submergence^42^, and other global studies.

Intertidal Habitat	Mean Reported Flux (µmolm^-2^h^-1^)			Flux measurement period	Reference
	CO_2_*	CH_4_	N_2_O		
Intertidal unvegetated flat (during emergence)	**−172** ± **76**	**0.05** ± **0.14**	**0.006** ± **0.015**	**~2–3 hour**	**This study**
	−1967 ± 1656			20–35 minute	^55^
	−2267 ± 1070			5 minute	^11^
			−0.29 ± 1.2	sediment core	^4^
			−0.25 ± 0.7	1.5 hour benthic	^10^
		5.3 ± 42		2 hour	^39^
Intertidal unvegetated flat (during submergence)			1.5 ± 3.7	1.5 hour	^10^
		0.14 ± 0.06	0.29 ± 0.19	5 hour	^2^
			0.36 ± 1.13	Meta analysis of 10 studies	^3^
	−974 ± 870			4 hour	^11^
	−1110 ± 636			6 hour	^9^
	**110** ± **2386**				^42^
Seagrass		48 ± 142		24 hour sediment cores	^59^
		11 ± 6		6 hour	^60^
	−5419 ± 3785			4 hour	^11^
			0.705 ± 1.12	Meta-analysis of 17 studies	^3^
Mangroves	206 ± 146			4 hour	^61^
	−1258 ± 3620	74 ± 705	2.4 ± 3.0	Meta-analysis of 18 studies	^62^
			0.44 ± 2.5	Meta-analysis of 17 studies	^3^
Saltmarsh		3.5 ± 4.1		Meta-analysis	^63^
	3122 ± 4302	2		Meta-analysis	^64^
			4.15 ± 7.65	1.5 hour	^10^
			2.8 ± 6.3	3 hour	^65^

^*^Average CO_2_ fluxes reported from measurements under light conditions.

Our study showed that GHG fluxes from intertidal habitats are lower per m^2^ than other coastal habitats, such as salt marsh, mangroves, or seagrass (Table 5). However, intertidal sandflats often cover vast areas within estuaries^57^, and in these instances the contribution of intertidal flats to estuarine GHG flux fluxes is substantial. To demonstrate this, we calculate the contribution of intertidal flats to estuarine GHG emissions using a typical estuary from northern New Zealand as a case study (Tairua Harbour). After accounting for the extent of intertidal habitats within the estuary (33% based on habitat maps reported in Needham *et al*.^58^), we calculate that unvegetated, intertidal flats account for approximately 8% of the total CO_2_ emissions within estuaries, 1% of the CH_4_ emissions, and 24% of the N_2_O emissions (Table 6).

**Table 6:** Fluxes of GHGs in Tairua Harbour.

Habitat	Area (m^2^)	% of total estuary	Mean net flux (µmol m^−2^ h^−1^)			Annual net flux (mol yr^−1^) in Tairua			Proportion of flux from each habitat		
			CO_2_	CH_4_	N_2_O	CO_2_	CH_4_	N_2_O	CO_2_	CH_4_	N_2_O
Intertidal unvegetated flat	3,200,000	48%	530^[1,2]^	0.095^[1,3]^	0.36^[1,3,5,13]^	1697	0.304	1.155	0.08	0.01	**0.24**
Subtidal unvegetated flat	1,315,000	20%	8060^[2]^	7.2^[4]^	0.81^[5]^	10600	9.468	1.065	0.52	0.18	0.22
Mangroves	361,000	5%	22917^[6]^	74^[7]^	0.44^[8]^	8273	26.71	0.1588	0.40	0.51	0.03
Seagrass	1,307,000	20%	−1041^[9]^	11^[10]^	0.71^[5]^	−1361	14.38	0.9214	−0.07	0.27	0.19
Salt Marsh	417,000	6%	3122^[11]^	3.5^[12]^	3.48^[13,14]^	1302	1.460	1.451	0.06	0.03	0.31
Total	6,600,000	100%				20510	52.32	4.752	1	1	1

Areas are based of the mapping carried out by Needham *et al*.^58^. Where possible, fluxes from New Zealand studies were used. When that data was not available, fluxes from estuaries at similar latitudes were used. Mean fluxes are the mean net flux throughout a day, and account for tidal and diurnal variation, including fluxes between emerged and submerged periods, and (where appropriate) photosynthetically active and dark conditions.1 - This study; 2 – Thrush *et al*., submitted; 3 - Li *et al*., 2019; 4 - Abril & Borges, 2004; 5 – Murray, Erler & Eyre, 2015; 6 - Bulmer *et al*., 2017; 7 - Chen *et al*., 2010; 8 - Livesly & Andrusiak, 2012; 9 - Drylie *et al*., 2017; 10 - Bahlmann, 2015; 11 - Lovelock *et al*., 2017; 12 - Poffenberger *et al*., 2001; 13 - Wang *et al*., 2006; 14 - Tang, 2016.

## Conclusions

This study has shown that nutrient enrichment increases GHG fluxes from emerged intertidal flats (increasing CO_2_ uptake under light emissions, but also increasing the emission of CH_4_ and N_2_O). These results suggest that increased nitrogen enrichment of estuarine systems will likely lead to increased emission of GHGs, contributing to increased global GHG emissions and potentially exacerbating the impact of climate change. This study has also shown that emerged GHG fluxes from intertidal flats are an important component of estuarine GHG fluxes, particularly in estuaries where intertidal flats cover significant a significant proportion of the estuary. Finally, our study demonstrates that tidal state (submerged vs emerged) needs to be carefully considered when upscaling estimates of GHG emissions from intertidal flats, as directions and magnitudes of GHG fluxes may differ between tidal emergence and submergence.

## Supplementary information

Supplementary Information.
