# Supplementary material for: Nitrogen enrichment increases greenhouse gas emissions from emerged intertidal sandflats
Source: Sci Rep. 2020 Apr 21;10:6686. doi: 10.1038/s41598-020-62215-4 (PMC7174373; doi:10.1038/s41598-020-62215-4)
Supplement: Supplementary file 1 — Supplementary Information. [file 41598_2020_62215_MOESM1_ESM.docx]

## Supplementary Information

| Specification | N_2_O | CH_4_ | CO_2_ | NH_3_ | H_2_O |
| --- | --- | --- | --- | --- | --- |
| Precision Raw (1σ) | <25 ppb +0.05% of reading | <10 ppb +0.05% of reading | <600 ppb +0.05% of reading | <5 ppb +0.05% of reading | <500 ppm |
| Precision 1 min (1σ) | <10 ppb +0.05 % of reading | <7 ppb +0.05 % of reading | <300 ppb +0.05 % of reading | <3 ppb +0.05 % of reading | <250 ppm |
| Precision 5 min (1σ) | <5 ppb +0.008 % of reading | <5 ppb +0.02 % of reading | <200 ppb +0.05 % of reading | <1 ppb +0.05 % of reading | <100 ppm |
| Guaranteed Spec Range | 0.3-200 ppm | 1.5-12 ppm | 380-5000 | 0-300 ppb | 0-3 % |
| Operating Range | 0-400 ppm | 0.5-15 ppm | 0.02-2 % | 0-2 ppm | 0-7 % |
| Measurement Rate | <8 seconds | <8 seconds | <8 seconds | <8 seconds | <8 seconds |
| Typical Gas Response (Rise-Fall 10-90%, 90-10%) | ~8 seconds | ~8 seconds | ~8 seconds |  |  |
| Report Dry Mole Fraction | Yes | Yes | Yes | No | N/A |

**Table S.1.** Picarro G2508 Gas Analyser performance specifications in air.
